# Longitudinal trends in blood pressure, prevalence, awareness, treatment, and control of hypertension in the Czech population. Are there any sex differences?

**DOI:** 10.3389/fcvm.2022.1033606

**Published:** 2022-11-10

**Authors:** Renata Cífková, Jan Bruthans, Larysa Strilchuk, Peter Wohlfahrt, Alena Krajčoviechová, Pavel Šulc, Marie Jozífová, Lenka Eremiášová, Jan Pudil, Aleš Linhart, Jiří Widimský, Jan Filipovský, Otto Mayer, Zdenka Škodová, Věra Lánská

**Affiliations:** ^1^Center for Cardiovascular Prevention, First Faculty of Medicine and Thomayer University Hospital, Charles University in Prague, Prague, Czechia; ^2^Department of Medicine II, First Faculty of Medicine, Charles University in Prague, Prague, Czechia; ^3^Department of Therapy No. 1, Medical Diagnostics, Hematology and Transfusiology, Danylo Halytsky Lviv National Medical University, Lviv, Ukraine; ^4^Department of Medicine III, First Faculty of Medicine, Charles University, Prague, Czechia; ^5^Department of Medicine II, Faculty of Medicine, Charles University, Pilsen, Czechia; ^6^Department of Preventive Cardiology, Institute for Clinical and Experimental Medicine, Prague, Czechia; ^7^Medical Statistics Unit, Institute for Clinical and Experimental Medicine, Prague, Czechia

**Keywords:** Czech MONICA, Czech post-MONICA study, epidemiology of hypertension, population random sample, response rate

## Abstract

**Background:**

Hypertension is the most common cardiovascular disease which substantially increases cardiovascular morbidity and mortality. Despite the broad availability of antihypertensive medication, control of hypertension is not satisfactory worldwide.

**Objective:**

The study aim was to assess longitudinal trends in blood pressure, prevalence, awareness, treatment, and control of hypertension in a representative population sample of the Czechia from 1985 to 2016/2017, focusing on sex differences.

**Methods:**

A total of 7,606 men and 8,050 women aged 25–64 years were screened for major CV risk factors in seven independent cross-sectional surveys run consistently in the same six country districts of the Czechia between 1985 and 2016/2017. The population samples were randomly selected.

**Results:**

Over a study period of 31/32 years, there was a significant decline in systolic and diastolic blood pressure in both sexes, whereas the prevalence of hypertension decreased only in women. There was an increase in hypertension awareness in both sexes over the entire study period with consistently higher rates in women. The proportion of individuals treated with antihypertensive drugs increased significantly in both sexes throughout the study, again with consistently higher rates in women. Control of hypertension increased significantly over the study period with consistently higher rates in women. The age-adjusted trends in blood pressure, prevalence, awareness, and treatment of hypertension were significantly different in men and women, always in favor of women. The age-adjusted trends in control of hypertension in treated patients were equally poor in both sexes.

**Conclusion:**

There are significant differences in longitudinal trends in blood pressure, prevalence, awareness, treatment, and control of hypertension between men and women, always in favor of women except for the control of hypertension in treated patients, where it is equally poor in both sexes.

## Introduction

Hypertension is the most prevalent cardiovascular disease affecting 30 – 50% of the adult population worldwide, with significant regional differences (1). Hypertension is also a major risk factor for developing stroke, coronary heart disease, heart and renal failure, peripheral arterial disease, aortic aneurysm, atrial fibrillation, and cognitive dysfunction/dementia (2, 3). Large clinical trials have convincingly shown that treatment of hypertension is followed by a decrease in cardiovascular morbidity and mortality (4, 5).

Hypertension can be easily detected and treated in primary care facilities. However, control of hypertension remains a major challenge throughout the global population. The Global Burden of Disease Study 2019 identified high systolic blood pressure (BP) (defined as a theoretical minimum risk exposure level of ≥110 – 115 mm Hg) as the leading Level 2 risk factor for death worldwide (3, 6).

The burden of hypertension can be reduced by simultaneously approaching the reduction of hypertension prevalence through primary prevention, and by increasing treatment and control of hypertension.

This analysis aimed to assess the longitudinal trends in BP, prevalence, awareness, treatment, and control of hypertension in a representative population sample of the Czechia from 1985 to 2016/2017, focusing on sex differences.

## Materials and methods

### Study population

A total of 7,606 men and 8,050 women aged 25–64 years were screened for major CV risk factors in seven independent cross-sectional surveys run in the same six country districts of the Czechia between 1985 and 2016/2017. The initial three surveys in 1985, 1988, and 1992 were conducted within the WHO MONICA Project (7), further referred to as the Czech MONICA Study.

The study population was always randomly selected as a one percent population sample within each district, stratified by age and sex, within an age range of 25–64 years. The details are published elsewhere (8).

The Czech MONICA and Czech post-MONICA studies (the last four surveys) were approved by the Ethics Committee of the Institute for Clinical and Experimental Medicine and Thomayer Hospital, Prague, Czechia. All participants provided informed consent.

### Screening examination

The methods used were detailed elsewhere (9). In short, the examination consisted of a questionnaire which was completed by a physician. Currently prescribed drugs were recorded and verified (when possible) against drug containers.

Height and body weight measurements were taken in the standing position without shoes and outer garments. BP measurement was performed consistently on the right arm (supported at the heart level), in the sitting position, after a minimum 5-min rest, using standard mercury sphygmomanometers and properly sized cuffs. Blood pressure values were recorded to the nearest 2 mmHg. In 1985, 1988, and 1992, two consecutive BP measurements were performed with their mean values used for the longitudinal trend analysis. In 1997/98, 2000/01, 2007/08, and 2016/17 the study protocol was extended by including three consecutive BP measurements; however, for the purpose of longitudinal trend analysis, only the mean of the first two readings was used.

### Definition of obesity and hypertension

We defined obesity as body mass index (BMI) ≥30 kg/m^2^ for both sexes.

Hypertension was defined as a mean SBP ≥140 mmHg, and/or a mean DBP ≥90 mmHg, or current treatment with antihypertensive drugs. Study participants who reported previously diagnosed hypertension or current use of antihypertensive medication were considered aware of their hypertension. Treatment of hypertension was defined as the current use of prescribed BP lowering medication. Control of hypertension was defined as a proportion of individuals with hypertension achieving both SBP <140 mmHg and DBP < 90 mmHg. We also provide data on the control of hypertension in drug-treated hypertensives, defined as the proportion of drug-treated hypertensive individuals achieving both SBP <140 mmHg and DBP <90 mmHg.

### Statistical analysis

Statistical analyses were performed using JMP^®^ 15.2.0 statistical software (2019, SAS Institute Inc.). Trends for means were tested by linear contrast in one-way ANOVA, and trends for percentage by Cochran Armitage trend test of proportions. ANCOVA and logistic regression with an interaction of sex and the year of examination were used to determine a possible influence of sex on trends, and the year(s) of examination on tested variables. When necessary, Bonferroni correction for the adjustment of p values was applied. All p values are two-sided and p < 0.05 is considered statistically significant.

## Results

### Population sample characteristics

**FIGURE 1:**
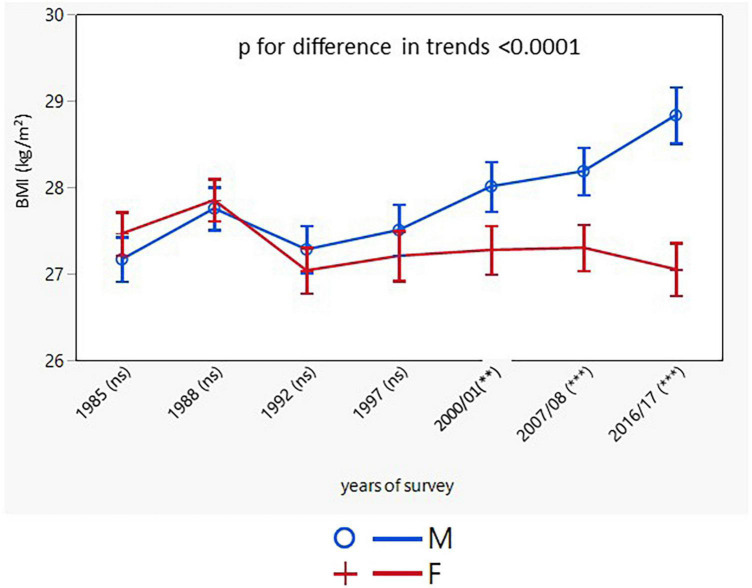
Age-adjusted trends in BMI (95% confidence interval). BMI, body mass index; M, males; F, females; *p* for differences in trends between males and females. NS or asterisks in brackets after survey years indicates the sex differences in respective surveys after Bonferroni correction; **p < 0.01; ***p < 0.001.

A total of 15,656 Caucasians participated in seven independent cross-sectional surveys (Table 1). The response rates showed a significant downward linear trend in both sexes, with a sharp decrease in the most recent survey, particularly in the youngest age groups. Women consistently had higher response rates than men throughout all age groups and surveys. Over the entire study period of 31–32 years, BMI significantly increased in men in all age groups, whereas BMI in women did not change and even declined in the age group of 45–54 years. Adjusted for age, the longitudinal trends in BMI in men and women differed (*p* < 0.0001) (Figure 1). Between 1985 and 1997/1998 trends in BMI in both sexes remained similar. In the 2001 survey, a sharp increase in BMI in men occurred and continued, whereas women’s BMI remained largely unchanged.

**TABLE 1:** Survey sample sizes, response rates, BMI, and obesity prevalence by sex and year of survey.

	1985	1988	1992	1997/98	2000/01	2007/08	2016/17	*p* for trend
**Total**	**2,570**	**2,768**	**2,343**	**1,990**	**2,055**	**2,246**	**1,684**	
Age, yrs (mean ± SD)	44.9 ± 11.38	45.1 ± 11.26	44.7 ± 10.87	45.6 ± 10.64	46.2 ± 11.9	47.1 ± 11.46	47.8 ± 10.85	<0.001
**Men**	**1,253**	**1,357**	**1,134**	**969**	**1,003**	**1,102**	**788**	
Age, years (mean ± SD)	45.0 ± 11.39	45.3 ± 11.29	44.6 ± 10.76	45.8 ± 10.63	46.7 ± 11.07	47.9 ± 11.65	48.0 ± 10.83	<0.001
Response rate (%)	81.5	85.5	73.2	63.2	62.0	62.1	43.1	<0.001
Age group, *n* (%)								
25–34	307 (24.5)	322 (23.7)	246 (21.7)	194 (20.0)	187 (18.6)	208 (18.9)	116 (14.7)	<0.001
35–44	296 (23.6)	323 (23.8)	350 (30.9)	230 (23.7)	230 (22.9)	251 (22.8)	198 (25.1)	ns
45–54	334 (26.7)	361 (26.6)	310 (27.3)	332 (34.3)	295 (29.4)	231 (21.0)	210 (26.7)	ns
55–64	316 (25.2)	351 (25.9)	228 (20.1)	213 (22.0)	291 (29.0)	412 (37.4)	264 (33.50)	ns
BMI, kg/m^2^, (mean ± SD)								
Total, 25–64 y	27.0 ± 4.0	27.7 ± 3.8	27.1 ± 3.8	27.5 ± 3.8	28.1 ± 4.4	28.5 ± 4.6	29.2 ± 5.1	0.001
25–34 y	25.5 ± 3.4	26.2 ± 3.3	25.2 ± 3.2	25.9 ± 3.2	26.2 ± 4.3	26.3 ± 4.3	27.5 ± 4.9	0.011
35–44 y	26.8 ± 3.8	27.1 ± 3.7	26.8 ± 3.6	26.7 ± 3.3	27.6 ± 3.9	28.0 ± 4.5	28.2 ± 4.8	<0.001
45–54 y	27.7 ± 3.8	28.1 ± 3.7	27.8 ± 3.6	28.2 ± 3.9	28.5 ± 4.2	28.7 ± 4.4	29.5 ± 5.0	<0.001
55–64 y	28.1 ± 4.3	29.2 ± 3.9	28.6 ± 4.1	28.8 ± 3.8	29.5 ± 4.6	29.8 ± 4.6	30.4 ± 5.3	<0.001
BMI ≥ 30 kg/m^2^, *n* (%)	246 (19.7)	343 (25.3)	225 (19.9)	244 (25.2)	295 (29.5)	370 (33.6)	297 (37.7)	<0.001
**Women**	**1,317**	**1,411**	**1,209**	**1,021**	**1,052**	**1,144**	**896**	
Age, years (mean ± SD)	44.9 ± 11.38	44.9 ± 11.24	44.9 ± 10.97	45.3 ± 10.65	45.8 ± 11.10	46.4 ± 11.23	47.6 ± 10.88	<0.001
Response rate (%)	85.0	88.4	76.7	66.4	63.8	63.1	48.6	<0.001
Age group, *n* (%)								
25–34	322 (24.4)	342 (24.2)	266 (22.0)	212 (20.8)	213 (20.2)	235 (20.5)	147 (16.4)	<0.001
35–44	340 (25.8)	369 (26.2)	356 (29.4)	266 (26.1)	276 (26.2)	284 (24.8)	204 (22.8)	ns
45–54	343 (26.0)	360 (25.5)	311 (25.7)	326 (31.9)	285 (27.1)	299 (26.1)	282 (31.5)	ns
55–64	312 (23.7)	340 (24.1)	276 (22.8)	217 (21.3)	278 (26.4)	326 (28.5)	263 (29.4)	ns
BMI, kg/m^2^, (mean ± SD)								
Total, 25–64 y	27.3 ± 5.4	27.7 ± 5.4	26.9 ± 5.3	27.1 ± 5.5	27.3 ± 5.7	27.3 ± 5.7	27.3 ± 6.0	ns
25–34 y	23.9 ± 4.1	24.3 ± 3.9	23.6 ± 4.0	24.2 ± 4.6	23.8 ± 4.1	23.8 ± 4.8	24.7 ± 5.4	ns
35–44 y	26.5 ± 4.7	26.9 ± 4.9	25.8 ± 4.9	25.8 ± 4.9	26.4 ± 5.5	26.6 ± 5.7	26.5 ± 6.0	ns
45–54 y	28.6 ± 4.9	29.0 ± 5.0	28.3 ± 5.5	28.4 ± 5.6	27.7 ± 5.1	27.9 ± 5.7	27.4 ± 5.6	0.007
55–64 y	30.4 ± 5.4	30.7 ± 5.4	29.9 ± 5.1	29.7 ± 5.0	30.4 ± 5.9	30.2 ± 5.9	29.4 ± 6.2	ns
BMI ≥ 30 kg/m^2^, *n* (%)	367 (28.0)	423 (30.0)	308 (25.5)	270 (26.5)	292 (27.8)	344 (28.1)	247 (27.6)	ns

BMI, body mass index; SD, standard deviation.

### Longitudinal trends in blood pressure and the prevalence, awareness, treatment, and control of hypertension

**FIGURE 2:**
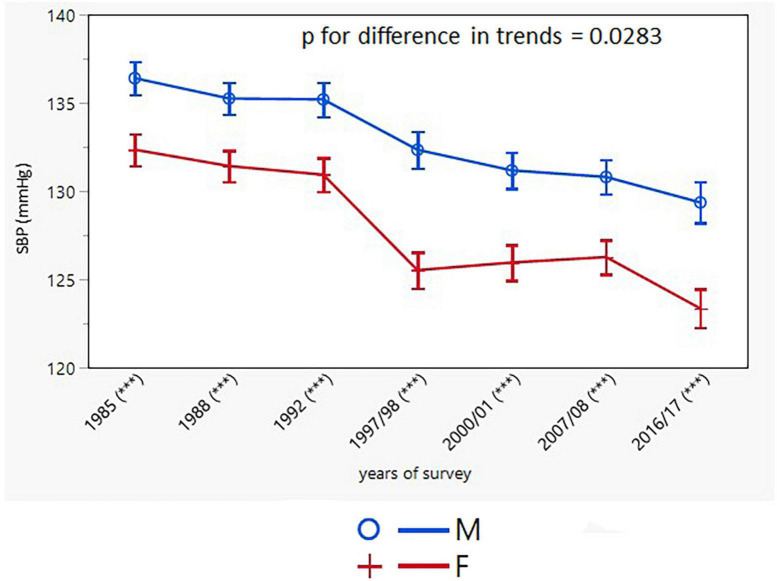
Age-adjusted trends in systolic blood pressure (95% confidence interval). M, males; F, females; *p* for differences in trends between males and females. Asterisks in brackets after survey years indicates the sex differences in respective surveys after Bonferroni correction; ***p < 0.001.

**FIGURE 3:**
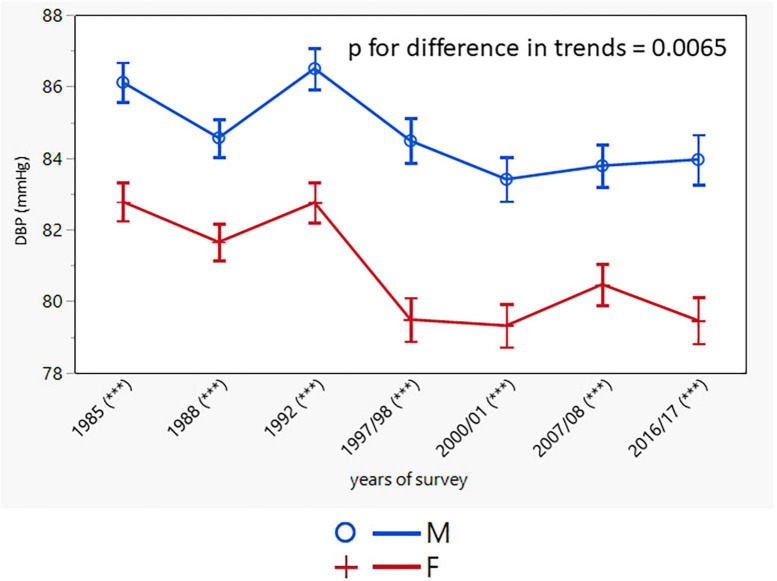
Age-adjusted trends in diastolic blood pressure (95% confidence interval). M, males; F, females; *p* for differences in trends between males and females. Asterisks indicates in brackets after survey years the sex differences in respective surveys after Bonferroni correction; ***p < 0.001.

SBP and DBP declined in both sexes with a greater decline in women (men: from 135.8 ± 19.2/85.9 ± 11.0 to 131.1 ± 14.9/84.7 ± 9.1 mmHg; *p* < 0.001; women: from 131.6 ± 20.9/82.5 ± 11.3 to 124.8 ± 16.9/80.0 ± 9.4 mmHg; *p* < 0.001) (Table 2). SBP and DBP values in the two youngest male age groups did not change, whereas in women no change was observed only for DBP in the youngest age group. The trends in SBP and DBP, adjusted for age, were different in men and women, with BP values remaining constantly higher in men over the entire study period (Figures 2, 3).

**TABLE 2:** Blood pressure (mean ± SD) between 1985 and 2016/17 in six districts of the Czechia.

	1985	1988	1992	1997/98	2000/01	2007/08	2016/17	*p* for trend
**Men**								
SBP, mmHg								
Total, 25–64 y	135.8 ± 19.2	134.9 ± 19.2	134.2 ± 20.0	132.3 ± 16.9	131.9 ± 16.8	132.5 ± 17.3	131.1 ± 14.9	<0.001
25–34 y	125.7 ± 14.6	125.5 ± 13.6	123.9 ± 13.4	124.3 ± 11.4	125.0 ± 15.3	124.8 ± 11.7	124.9 ± 12.6	ns
35–44 y	129.9 ± 15.7	128.1 ± 15.5	128.2 ± 15.4	127.9 ± 13.7	126.4 ± 13.9	127.6 ± 13.9	127.1 ± 13.1	ns
45–54 y	139.9 ± 18.9	137.7 ± 19.1	140.5 ± 20.1	132.4 ± 15.9	132.8 ± 16.0	131.3 ± 16.6	131.6 ± 14.2	<0.001
55–64 y	146.7 ± 19.4	146.7 ± 19.9	146.0 ± 22.8	144.3 ± 19.1	139.7 ± 17.1	139.9 ± 18.7	136.5 ± 15.6	<0.001
DBP, mmHg								
Total, 25–64 y	85.9 ± 11.0	84.4 ± 11.0	86.1 ± 11.4	84.5 ± 10.0	83.7 ± 9.7	84.4 ± 10.1	84.7 ± 9.1	<0.001
25–34 y	81.1 ± 10.0	79.9 ± 10.3	80.8 ± 9.7	80.0 ± 8.7	79.7 ± 9.7	79.9 ± 8.5	81.2 ± 9.6	ns
35–44	84.8 ± 9.8	82.7 ± 10.3	84.9 ± 10.1	83.2 ± 9.5	82.7 ± 9.2	84.1 ± 10.4	84.0 ± 8.5	ns
45–54 y	88.7 ± 11.2	86.5 ± 11.1	86.4 ± 11.4	86.4 ± 9.6	85.2 ± 9.2	85.2 ± 8.9	85.8 ± 9.2	<0.001
55–64 y	88.5 ± 11.2	87.9 ± 10.4	89.3 ± 11.7	87.0 ± 10.8	85.5 ± 9.6	86.5 ± 10.6	85.7 ± 8.9	<0.001
**Women**								
SBP, mmHg								
Total, 25–64 y	131.6 ± 20.9	130.7 ± 20.9	130.2 ± 22.0	125.2 ± 18.1	125.9 ± 18.8	126.7 ± 19.2	124.8 ± 16.9	<0.001
25–34 y	116.6 ± 13.7	116.0 ± 12.2	115.6 ± 13.3	113.7 ± 9.9	112.9 ± 11.0	114.5 ± 12.2	114.7 ± 12.4	0.004
35–44 y	125.8 ± 15.8	124.3 ± 16.0	121.1 ± 16.0	118.9 ± 14.6	118.7 ± 12.7	120.1 ± 15.5	119.2 ± 13.4	<0.001
45–54 y	136.0 ± 19.1	135.7 ± 18.4	137.1 ± 21.0	129.0 ± 17.9	128.9 ± 17.8	128.6 ± 17.0	125.1 ± 16.3	<0.001
55–64 y	148.6 ± 19.9	147.1 ± 21.7	148.0 ± 21.2	138.2 ± 18.3	140.2 ± 19.1	139.5 ± 20.2	134.4 ± 17.0	<0.001
DBP, mmHg								
Total, 25–64 y	82.5 ± 11.3	81.4 ± 11.2	82.5 ± 12.1	79.3 ± 9.8	79.3 ± 9.8	80.6 ± 9.6	80.0 ± 9.4	<0.001
25–34 y	74.8 ± 9.2	74.4 ± 8.7	75.0 ± 9.1	73.8 ± 7.7	73.5 ± 7.9	75.7 ± 8.5	75.9 ± 9.3	ns
35–44 y	81.4 ± 9.9	79.1 ± 10.1	79.0 ± 10.5	77.1 ± 9.0	76.9 ± 8.8	79.3 ± 8.8	78.2 ± 9.0	<0.001
45–54 y	85.1 ± 10.7	84.5 ± 10.5	86.9 ± 11.9	81.7 ± 10.0	80.9 ± 8.8	82.0 ± 9.3	81.0 ± 9.2	<0.001
55–64 y	88.7 ± 10.3	87.6 ± 10.6	89.1 ± 11.5	83.9 ± 8.8	84.5 ± 10.0	84.2 ± 9.3	82.6 ± 8.8	<0.001

*p*, statistical significance for linear trend. SBP, systolic blood pressure, DBP, diastolic blood pressure.

**FIGURE 4:**
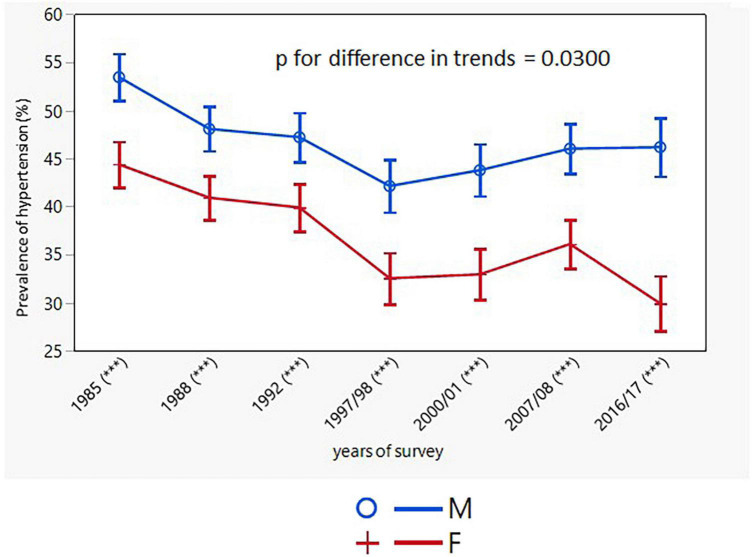
Age-adjusted trends in prevalence of hypertension (95% confidence interval). M, males; F, females; *p* for differences in trends between males and females. Asterisks indicates in brackets after survey years the sex differences in respective surveys after Bonferroni correction; ***p < 0.001.

The prevalence of hypertension declined only in women (from 42.5% in 1985 to 33.5% in 2016/17; *p* < 0.001; the decrease was significant in all age groups, except for the youngest one). In the male study population, there was no change in the prevalence of hypertension, except for two middle-aged groups (35–44 and 45–54 years) (Table 3). The age-adjusted trends in the prevalence of hypertension were different for men and women with consistently higher rates in men (Figure 4).

**TABLE 3:** Prevalence, awareness, and treatment of hypertension between 1985 and 2016/17 in six districts of the Czechia.

	1985	1988	1992	1997/98	2000/01	2007/08	2016/17	*p* for trend
**Men**								
Prevalence of HT, %								
Total, 25–64 y, *n* (%)	650 (51.9)	639 (47.1)	508 (44.8)	408 (42.1)	457 (45.6)	553 (50.2)	399 (50.6)	Ns
25–34 y	85 (27.7)	67 (20.8)	46 (18.7)	35 (18.0)	34 (18.2)	43 (20.7)	26 (22.4)	0.0730
35–44 y	123 (41.6)	119 (36.8)	126 (36.0)	61 (26.5)	69 (30.0)	82 (32.7)	66 (33.3)	0.049
45–54 s	208 (62.3)	198 (54.9)	186 (60.0)	161 (48.5)	147 (49.8)	122 (52.8)	107 (51.0)	0.009
55–64 y	234 (74.1)	255 (72.7)	150 (65.8)	151 (70.9)	207 (71.1)	306 (74.3)	200 (75.8)	Ns
Awareness of HT, *n* (%)								
Total, 25–64 y	269 (41.4)	320 (50.1)	232 (45.7)	230 (56.4)	284 (62.1)	378 (68.4)	290 (74.6)	<0.001
25–34 y	23 (27.1)	27 (40.3)	17 (37.0)	12 (34.3)	13 (38.2)	13 (30.2)	15 (62.5)	Ns
35–44 y	38 (30.9)	53 (44.5)	52 (41.3)	29 (47.5)	37 (53.6)	45 (54.9)	45 (68.2)	<0.0001
45–54 y	91 (43.8)	87 (43.9)	82 (44.1)	88 (54.7)	91 (61.9)	82 (67.2)	69 (64.5)	<0.0001
55–64 y	117 (50.0)	153 (60.0)	81 (54.0)	101 (66.9)	143 (69.1)	238 (77.8)	161 (80.9)	<0.0001
Medication for HT, *n* (%)								
Total, 25–64 y	137 (21.1)	197 (30.8)	123 (24.2)	151 (37.0)	191 (41.8)	322 (58.2)	241 (60.9)	<0.001
25–34 y	3 (3.5)	8 (11.9)	1 (2.2)	6 (17.1)	3 (8.9)	4 (9.3)	4 (15.4)	0.0909
35–44 y	16 (13.0)	22 (18.5)	20 (15.9)	11 (18.0)	17 (24.6)	26 (31.7)	31 (47.0)	<0.0001
45–54 y	50 (24.0)	55 (27.8)	54 (29.0)	58 (36.0)	62 (42.2)	72 (59.0)	57 (53.3)	<0.0001
55–64 y	68 (29.1)	112 (43.9)	48 (32.0)	76 (50.3)	109 (52.7)	220 (71.9)	149 (74.5)	<0.0001
**Women**								
Prevalence of HT, *n* (%)								
Total, 25–64 y	560 (42.5)	552 (39.1)	460 (38.0)	323 (31.6)	347 (33.0)	426 (37.3)	300 (33.5)	<0.001
25–34 y	30 (9.3)	27 (7.9)	22 (8.3)	7 (3.3)	10 (4.7)	16 (6.8)	7 (4.8)	ns
35–44 y	98 (28.8)	88 (23.9)	67 (18.8)	41 (15.4)	37 (13.4)	62 (21.9)	26 (12.8)	<0.001
45–54 y	186 (54.2)	180 (50.0)	166 (53.4)	134 (41.1)	110 (38.6)	124 (41.5)	111 (39.4)	<0.001
55–64 y	246 (78.9)	257 (75.6)	205 (74.3)	141 (65.0)	190 (68.4)	224 (68.7)	156 (59.3)	<0.001
Awareness of HT, *n* (%)								
Total, 25–64 y	330 (58.9)	330 (59.8)	255 (55.4)	221 (68.4)	256 (73.8)	304 (71.4)	233 (77.7)	<0.001
25–34 y	14 (46.7)	15 (55.6)	6 (27.3)	1 (14.3)	4 (40.0)	9 (56.3)	6 (85.7)	ns
35–44 y	40 (40.8)	42 (47.7)	30 (44.8)	27 (65.9)	26 (70.3)	36 (58.0)	19 (73.1)	0.0001
45–54 y	109 (58.6)	105 (58.3)	91 (54.8)	83 (61.9)	72 (65.5)	94 (75.8)	78 (70.3)	0.0003
55–64 y	167 (67.9)	168 (65.4)	128 (62.4)	110 (78.0)	154 (81.1)	165 (73.7)	130 (83.3)	<0.0001
Medication for HT, *n* (%)								
Total, 25–64 y	218 (38.9)	233 (42.2)	159 (34.6)	187 (57.9)	205 (59.1)	251 (58.9)	193 (64.8)	<0.001
25–34 y	5 (16.7)	5 (18.5)	2 (9.1)	0 (0.0)	1 (10.0)	4 (25.0)	1 (14.3)	ns
35–44 y	13 (13.27)	24 (27.3)	8 (11.9)	22 (53.4)	16 (43.2)	26 (41.9)	13 (50.0)	<0.0001
45–54 y	82 (44.1)	73 (40.6)	54 (32.5)	64 (47.8)	59 (53.6)	77 (62.1)	67 (60.4)	<0.0001
55–64 y	118 (48.0)	131 (51.0)	95 (46.3)	101 (71.6)	129 (67.9)	144 (64.3)	112 (71.8)	<0.0001

*p*, statistical significance for linear trend. SBP, systolic blood pressure, DBP, diastolic blood pressure, HT, hypertension.

**FIGURE 5:**
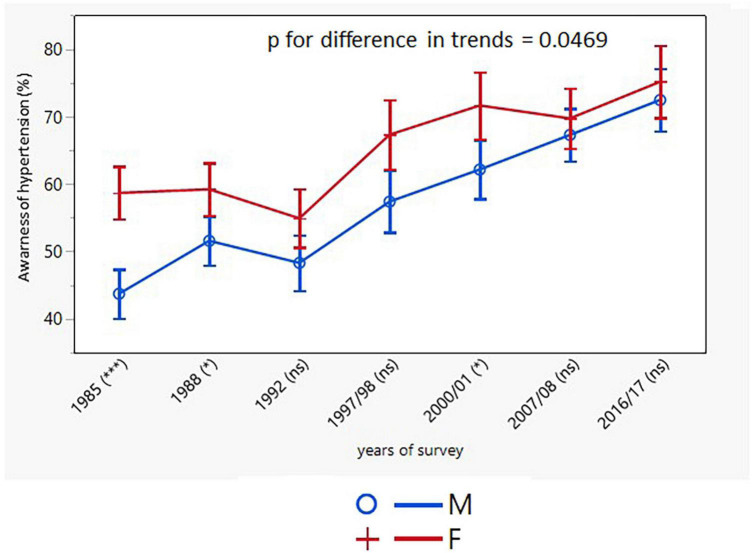
Age-adjusted trends in awareness of hypertension (95% confidence interval). M, males; F, females; *p* for differences in trends between males and females. NS or asterisks indicates in brackets after survey years the sex differences in respective surveys after Bonferroni correction; *p < 0.05; ***p < 0.001.

There was an increase in hypertension awareness in both sexes over the entire study period with consistently higher rates in women (men: from 41.4% in 1985 to 74.6% in 2016/17; *p* < 0.001; women: from 58.9% in 1985 to 77.7% in 2016/17; *p* < 0.001) (Table 3). However, there was no increase in awareness of hypertension in the youngest age groups of both sexes. The age-adjusted trends in awareness were different in men and women (Figure 5).

**FIGURE 6:**
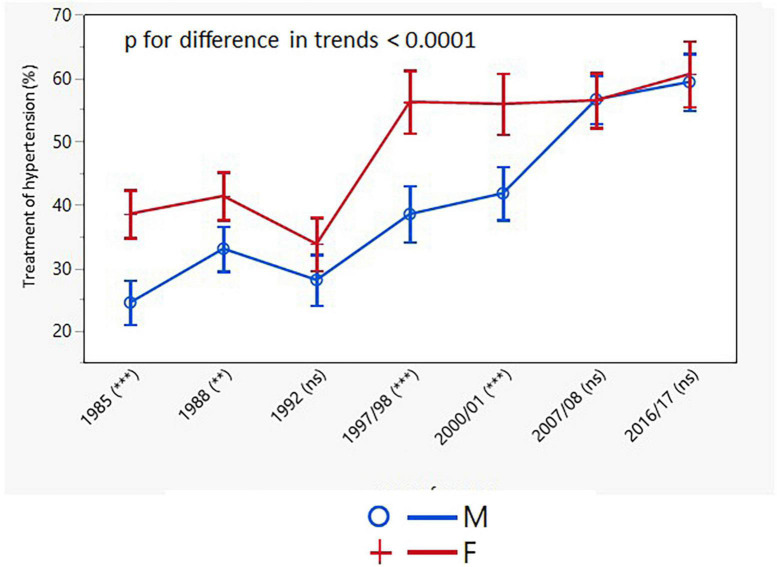
Age-adjusted trends in treatment of hypertension (95% confidence interval). M, males; F, females; *p* for differences in trends between males and females. NS or asterisks indicates in brackets after survey years the sex differences in respective surveys after Bonferroni correction; **p < 0.01; ***p < 0.001.

The proportion of individuals treated with antihypertensive drugs increased significantly in both sexes throughout the study, again with consistently higher rates in women (men: from 21.1 to 60.9%; *p* < 0.001; women: from 38.9 to 64.8%; *p* < 0.001) (Table 3). There was no change in the younger age groups of both sexes. The age-adjusted trends in the treatment of hypertension differed between men and women (Figure 6).

**FIGURE 7:**
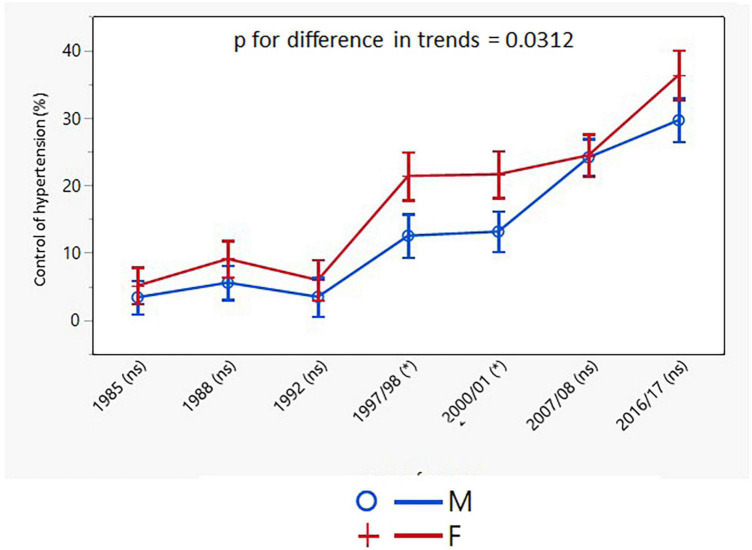
Age-adjusted trends in control of hypertension in all hypertensive individuals (95% confidence interval). M, males; F, females; *p* for differences in trends between males and females. NS or asterisks indicates in brackets after survey years the sex differences in respective surveys after Bonferroni correction; *p < 0.05.

**FIGURE 8:**
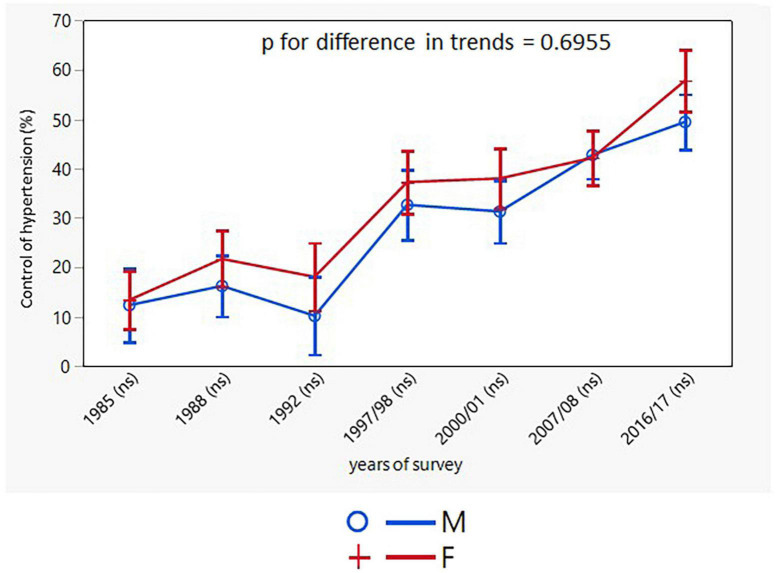
Age-adjusted trends in control of hypertension in drug treated hypertensives (95% confidence interval). M, males; F, females; *p* for differences in trends between males and females. NS indicates in brackets after survey years the sex differences in respective surveys after Bonferroni correction.

Control of hypertension increased significantly over the 30-year study period with consistently higher rates in women. The improvement in the control of hypertension was consistent in both sexes across all the age groups, except for the youngest female group (Table 4). The age-adjusted trends for control of hypertension were different for men and women only when control of hypertension was presented as the proportion of individuals with BP <140/90 mmHg in all hypertensive individuals, but not only in drug-treated hypertensive patients (Figures 7, 8).

**TABLE 4:** Control of hypertension between 1985 and 2016/17 in six districts of the Czechia.

	1985	1988	1992	1997/98	2000/01	2007/08	2016/17	*p* for trend
**Men**								
Control of hypertension (% of all hypertensive patients)								
Total, 25–64 y	18 (2.8)	33 (5.2)	14 (2.8)	50 (12.3)	60 (13.1)	135 (24.4)	119 (29.8)	<0.001
25–34 y	0 (0.0)	1 (1.5)	0 (0.0)	2 (5.7)	0 (0.0)	4 (9.3)	1 (3.9)	0.009
35–44 y	2 (1.6)	6 (5.0)	2 (1.6)	5 (8.2)	6 (8.7)	11 (13.4)	17 (25.8)	<0.0001
45–54 y	9 (4.3)	11 (5.6)	8 (4.3)	21 (13.0)	18 (12.2)	37 (30.3)	28 (26.2)	<0.0001
55–64 y	7 (3.0)	15 (5.9)	4 (2.7)	22 (14.6)	36 (17.4)	83 (27.1)	73 (36.5)	<0.0001
Control of hypertension (% of all drug-treated hypertensive patients)								
Total, 25–64 y	18 (13.1)	33 (16.8)	14 (11.4)	50 (33.1)	60 (31.4)	135 (41.9)	119 (49.4)	<0.0001
25–34 y	0 (0.0)	1 (12.5)	0 (0.0)	2 (33.3)	0 (0.0)	4 (100.0)	1 (25.0)	0.040
35–44 y	2 (12.5)	6 (27.3)	2 (10.0)	5 (45.5)	6 (36.3)	11 (42.3)	17 (54.8)	0.0005
45–54 y	9 (18.0)	11 (20.0)	8 (14.8)	21 (36.2)	18 (29.0)	37 (51.4)	28 (49.1)	<0.0001
55–64 y	7 (10.3)	15 (13.4)	4 (8.3)	22 (29.0)	36 (33.0)	83 (37.7)	73 (49.0)	<0.0001
**Women**								
Control of hypertension (% of all hypertensive patients)								
Total, 25–64 y	29 (5.2)	51 (9.2)	28 (6.1)	70 (21.7)	77 (22.2)	106 (24.9)	111 (37.0)	<0.001
25–34 y	0 (0.0)	2 (7.4)	1 (4.6)	0 (0.0)	1 (10.0)	1 (6.25)	0 (0.0)	ns
35–44 y	4 (4.1)	8 (9.1)	5 (7.5)	10 (24.4)	7 (19.0)	17 (27.4)	9 (34.6)	<0.0001
45–54 y	17 (9.1)	19 (10.6)	9 (5.4)	26 (19.4)	26 (23.6)	33 (26.6)	44 (39.6)	<0.0001
55–64 y	8 (3.3)	22 (8.6)	13 (6.3)	34 (24.1)	43 (22.6)	55 (24.6)	58 (37.2)	<0.0001
Control of hypertension (% of all drug-treated hypertensive patients)								
Total, 25–64 y	29 (13.3)	51 (21.9)	28 (17.6)	70 (37.4)	77 (37.6	106 (42.2)	111 57.5)	<0.0001
25–34 y	0 (0.0)	2 (40.0)	1 (50.0)	NA	1 (100.0)	1 (25.0)	0 (0.0)	ns
35–44 y	4 (30.8)	8 (33.3)	5 (62.5)	10 (45.5)	7 (43.8)	17 (65.4)	9 (69.2)	0.0065
45–54 y	17 (20.7)	19 (26.0)	9 (16.7)	26 (40.6)	26 (44.1)	33 (42.9)	44 (65.7)	<0.0001
55–64 y	8 (6.8)	22 (16.8)	13 (13.7)	34 (33.7)	43 (33.3)	55 (38.2)	58 (51.8)	<0.0001

*p*, statistical significance for linear trend; NA, not applicable as no individuals reported medication for hypertension.

## Discussion

The current analysis is, to the best of our knowledge, the first one assessing the longitudinal trends in BP, prevalence, awareness, treatment, and control of hypertension, paying special attention to sex differences. The main message is that there are significant differences, always in favor of women, in trends in all parameters listed above, except for in the control of hypertension in treated patients, where results were equally poor in both sexes. There are only a few countries with longitudinal data on the epidemiology of hypertension derived from representative population samples (USA, Canada, Korea, Sweden, Lithuania, and Finland) (10–15). Trends in BP and various aspects of the epidemiology of hypertension were described for several European countries but, as far as we know, none of the other studies specifically compared the trends in men and women.

### Strengths and limitations

The strength of our study is that it was always conducted in the same six districts of the Czechia using standardized methods, which were introduced by the WHO MONICA Project. The study protocol respected seasonal variations. The gold-standard mercury sphygmomanometer was used to measure BP in all seven cross-sectional surveys. The study period lasted 31/32 years, covering the transition from a totalitarian regime to democracy in the Czechia.

A decline in the response rate is a possible study limitation. This may have resulted in the population sample coming from a higher socio-economic background, which is usually associated with higher health consciousness. Therefore, our results might be slightly more favorable than in the actual general population. It should be noted that a decline in response rates has occurred in epidemiological studies worldwide, with recently reported rates below 40% (16–18). The European Health Examination Survey Pilot Project, conducted 2009-2012 in 12 countries involving individuals of the same age as our study population (25–64 years), found the participation rates to be 16-57% in men and 31–74% in women (16). The WHO MONICA Project examined non-respondents who were found more likely to be single, less educated, with poorer lifestyles, and worse health profiles than respondents (19).

### Trends in blood pressure and prevalence

A pooled analysis of 1,479 studies including 19.1 million adults found a decline in mean systolic and mean diastolic pressure from 1975 to 2015 in high-income western and high-income Asia Pacific super-regions (20). A later analysis by the same research group also reported a decrease in systolic BP in women in central and eastern Europe, but not in men (21).

An Austrian representative population-based study (*n* = 178,818), which included self-reported data from five health surveys between 1973 and 2007, showed that during the study period the age-standardized hypertension prevalence increased from 1.0 to 18.8%, with a considerable rise from 1991 onward. There was a positive trend in all subpopulations, especially in obese women (+ 50.2%) and obese individuals aged 75 years and older (+ 54.4%) (22).

Analysis of Lithuanian data from three MONICA health surveys (1983, 1986, and 1992) and one survey according to MONICA protocol (2002) concluded that during this period hypertension prevalence in men was 52.1 to 58.7% (no significant changes), whereas in women it decreased from 61.0 to 51.0% (15). The German National Health Interview and Examination Survey 1998 and 2008 to 2011 reported a decrease in age- and sex-standardized mean systolic and diastolic BP. The mean systolic and diastolic BP decrease was achieved in treated hypertensive patients, but there was also a BP decrease in normotensive individuals. The prevalence of hypertension increased only in men (29.8 to 33.4%; *p* < 0.03) (23).

In our study, there was a decline in the mean SBP and DBP in both sexes, however, the changes were more pronounced in women. The more favorable decline in BP in women can be partly explained by their BMI showing no significant change, whereas men’s BMI increased in all age groups over the entire study period.

The NCD Risk Factor Collaboration group reported a stable age-standardized prevalence of hypertension in adults aged 30-79 years a net effect of a decline in high-income countries and for women also in central and eastern Europe, and a rise in some low-income and middle-income countries. The results from our study mirrored these trends, with consistently lower rates and greater changes in women (Table 3 and Figure 4).

### Awareness, treatment, and control of hypertension

Awareness of hypertension dramatically improved throughout our study in both sexes, with women having started in a better position (58.9%) than men (41.4%). Both sexes achieved around 75% awareness by 2016/2017 (men 74.6%, women 77.7%), which is substantially better than in the NCD Risk Factor Collaboration report (globally, men 51%, women 41%). A similar increase in hypertension awareness was noted in Lithuania from 1983 to 2002 in both men and women (from 45.0 to 64.4% and 47.7 to 72.3%, respectively) and in treated hypertensives (from 55.4 to 68.3% in men and 65.6 to 86.2% in women) (15). Awareness and treatment of hypertension increased in Germany in both sexes (awareness: men from 65-78%, women from 74 to 87%; treatment: men from 48 to 65%, women from 62 to 79%) from 1998 to 2008 to 2011 (23).

Hypertension treatment and control have improved in most countries since 1990, with the greatest improvement in high-income countries and central Europe (21). The trends in treatment and hypertension control in the Czechia are in parallel with these findings. As with awareness of hypertension, women were treated for hypertension more frequently than men until the start of the millennium. Due to a greater increase in treatment of hypertension in men, no differences between the sexes were found in the last two surveys in the Czechia (final survey: men 60.9%, women 64.8%). On the other hand, in 2019 only 38% of men and 47% of women were treated globally (21).

Control of hypertension in the Czechia improved significantly in both men and women. The improvement mostly ran in parallel throughout the study, particularly in treated hypertensive patients. However, the final numbers for control of hypertension are still not sufficient (treated hypertensive patients: men 49.4%, women 57.5%). According to the NCD Risk Factor Collaboration report, the control rates in high-income countries rose to 60% (21). The Swedish primary care register shows that from 2010 to 2017 the proportion of patients with BP < 140/90 mmHg increased from 38.9 to 49.1% (24). Authors of the analysis of German Health Examination Surveys even claim that the control rate among treated hypertensives increased from 23% in 1998 to 51% in 2008 to 2011 (men from 20 to 45%, women from 25% to 58%) (23).

It is an alarming finding of our study that awareness, treatment, and control of hypertension did not improve in the youngest age group in both sexes. Hypertension was newly detected predominantly in young men with only a small proportion of them being treated for it. In the youngest age group hypertension was poorly controlled in both sexes. This is in concordance with other studies reporting poor levels of treatment and control in young adults compared with older adults (25–27).

It is a surprising fact that women who had lower SBP and DBP as well as a lower prevalence of hypertension were more frequently aware of the disease and were reported to be more frequently treated by antihypertensive drugs but they did not show better control of hypertension. This could be explained by less aggressive treatment in women or by their lower adherence to medication. Unfortunately, we have more precise data on the antihypertensive medication only for the last four surveys. Age-adjusted trends in hypertensive medication did not differ between men and women from 1997/98 to 2016/17, both increasing over time but staying significantly higher in men, meaning men took on average more antihypertensive drugs than women. Clearly, when we analyzed the proportion of monotherapy, a combination of two drugs, and a combination of three and more drugs, there was a significant increase over time favoring the triple and more combination in both sexes. However, this increase was steeper in men (in the last survey, 44.9% of men were treated with a combination of ≥3 drugs, compared to 36.1% of women). The less aggressive treatment of hypertension in women may correspond with the finding that women with coronary heart disease are less likely to be treated following the guidelines than men (28).

Another reason for women having unexpectedly equally poor control of their hypertension could be worse adherence to antihypertensive medication. This issue is controversial, depending on the method used to assess adherence. A meta-analysis of 82 studies which included 15,517,457 men and 18,537,599 women showed no significant differences in adherence between the sexes. However, this analysis was based on self-reported adherence and pharmacy refill records (29). On the other hand, studies on apparent treatment-resistant hypertension employing therapeutic drug monitoring showed that antihypertensive drug adherence was lower in women (30). Heterogeneity in published results must be acknowledged, partly due to various methods being used for assessing adherence.

## Conclusion and further perspective

We found significant differences in longitudinal age-adjusted trends in BP, prevalence, awareness, treatment, and control of hypertension between the sexes. The differences were always in favor of women except for control of hypertension in treated patients, which did not show any difference between men and women.

In conclusion, the trends in BP, prevalence, awareness, treatment, and control of hypertension in women showed similar pattern as women in high-income countries, whereas men are lagging in their awareness rates.

Future epidemiological studies in hypertension should also assess adherence to medication using objective methods rather than relying on self-reported data.

## Data availability statement

The datasets presented in this study can be found in online repositories. The names of the repository/repositories and accession number(s) can be found below: http://www.ftn.cz/data-monica-1117/.

## Ethics statement

The studies involving human participants were reviewed and approved by Ethics Committee of the Institute for Clinical and Experimental Research and Thomayer University Hospital Prague, Czechia. The patients/participants provided their written informed consent to participate in this study.

## Author contributions

All authors listed have made a substantial, direct, and intellectual contribution to the work, and approved it for publication.
